# Highlighting the Potency of Biosurfactants Produced by* Pseudomonas* Strains as Anti-*Legionella* Agents

**DOI:** 10.1155/2018/8194368

**Published:** 2018-10-22

**Authors:** Clémence Loiseau, Emilie Portier, Marie-Hélène Corre, Margot Schlusselhuber, Ségolène Depayras, Jean-Marc Berjeaud, Julien Verdon

**Affiliations:** ^1^Laboratoire Ecologie & Biologie des Interactions, UMR CNRS 7267, Université de Poitiers, France; ^2^Laboratoire Aliments Bioprocédés Toxicologie Environnements, EA 4651, Normandie Univ, UNICAEN, UNIROUEN, Caen, France; ^3^Laboratoire de Microbiologie Signaux et Microenvironnement, EA 4312, Université de Rouen, France

## Abstract

*Legionella pneumophila*, the causative agent of Legionnaires' disease, is a waterborne bacterium mainly found in man-made water systems in close association with free-living amoebae and multispecies biofilms.* Pseudomonas* strains, originating from various environments including freshwater systems or isolated from hospitalized patients, were tested for their antagonistic activity towards* L. pneumophila*. A high amount of tested strains was thus found to be active. This antibacterial activity was correlated to the presence of tensioactive agents in culture supernatants. As* Pseudomonas* strains were known to produce biosurfactants, these compounds were specifically extracted and purified from active strains and further characterized using reverse-phase HPLC and mass spectrometry methods. Finally, all biosurfactants tested (lipopeptides and rhamnolipids) were found active and this activity was shown to be higher towards* Legionella *strains compared to various other bacteria. Therefore, described biosurfactants are potent anti-*Legionella *agents that could be used in the water treatment industry although tests are needed to evaluate how effective they would be under field conditions.

## 1. Introduction

Bacteria of the* Legionella *genus are Gram-negative natural inhabitants of freshwater environments. Among the 65* Legionella* species referenced to date,* L. pneumophila* is the leading cause of severe pneumonia called Legionnaires' disease (LD). Furthermore the serogroup 1 is responsible for 82.9% of the cases in Europe [1] and for over 80% of the cases worldwide [2, 3].* L. pneumophila *can colonize man-made water settings from natural water sources, being now considered as an opportunistic plumbing pathogen. Through the literature, we can notice that LD outbreaks have been linked to a variety of water sources like cooling towers, drinking water supply systems, spa pools, and even street cleaning trucks [4–6]. Multiplication of* Legionella* in those artificial water systems is highly facilitated by temperatures around 35°C and factors such as water stagnation, poor maintenance, no or reduced water disinfection and the presence of free-living protozoa feeding on biofilms [7, 8]. Biofilms have been identified as an ecological key niche in which* L. pneumophila* survives and stays in wait for its natural hosts, amoebae, and ciliates [9]. Indeed, protozoan predators are the natural hosts of* L. pneumophila*, and humans are accidental hosts as judged by the evidence that only a single and recent case of probable human-to-human transmission has been reported to date [10]. Following the uptake of* L. pneumophila* by phagocytic cells, this bacterium avoids lysosome mediated degradation and forms a unique replication-permissive compartment within its host cell, called the* Legionella* containing vacuole (LCV) [11]. After replication, they are able to evade LCV, escape from the spent host cell, and disseminate in the environment looking for new suitable hosts (for recent review see [12]).

In order to survive in water sources,* L. pneumophila *is facing other biological challengers aside from protozoa as represented by others microbial inhabitants. In 2008, a study screened 80 aquatic bacterial strains including 5 referenced strains and showed that 66.2% displayed antagonistic activity against* L. pneumophila* [13]. Interestingly, among the* Pseudomonas* genus (representing 75% of all tested strains), 72% were active. However, to date, the antagonistic molecules of interest remain uncharacterized. On the other hand, several authors have shown that many* Pseudomonas *species can produce biosurfactants which are surface-active compounds. Those compounds known to display various functional properties like in structural biofilm formation/cells dispersion also exhibit particularly lytic and growth-inhibitory activities against a broad range of microorganisms, including viruses, mycoplasmas, bacteria, fungi, and oomycetes [14–16]. Among the wide range of structurally different biosurfactants that have been identified to date,* Pseudomonas* species can produce glycolipids and lipopeptides [17, 18]. Lipopeptides are constituted by a lipid tail linked to a short cyclic or linear peptide moiety. Based on differences in the length and composition of the lipid moiety as well as in the type, number and configuration of the amino acids in the peptide chain were initially classified into four groups: Amphisin, syringomycin, tolaasin, and viscosin [19]. However, several lipopeptides produced by* Pseudomonas* spp. which were characterized later displayed structural features which differ from these archetypes like putisolvins, syringofactins, and orfamides that extend the initial classification [20–22]. The first lipopeptide described in the literature and which remains the best known biosurfactant to date is surfactin produced by many* Bacillus *strains [23]. Members of the surfactin family are constituted of a heptapeptide moiety linked to a *β*-hydroxylated fatty acid to form a cyclic lactone ring and display antiviral and antibacterial activities [24]. Recently, a surfactin mixture produced by the strain* B. subtilis* AM1 was found active against various* Legionella* strains, including nonpneumophila,* pneumophila* serogroup 1, and* pneumophila* nonserogroup 1 strains [25]. To the best of our knowledge, this is the first time that such an activity was demonstrated and this study remains the only one published so far. This activity was striking as several reviews have highlighted a limited activity of many lipopeptides against Gram-negative bacteria [14, 15, 17]. However, lipopeptides produced by* Pseudomonas* and* Bacillus* species have not been tested extensively for activity against other saprophytic bacteria, but mostly for activity against human pathogenic bacteria like* Bacillus* sp. or* Mycobacterium* sp. [14]. The surfactin mixture from* B. subtilis* AM1 was also able to break down existing biofilms of* L. pneumophila* [25] suggesting that it could represent a potent tool for the biological control of the pathogen in the water treatment industry.

Rhamnolipids are glycolipid secondary metabolites typically constituted of dimer of 3-hydroxy fatty acids linked through a beta glycosidic bond to a mono- or di-rhamnose moiety [26]. Up to now, more than one hundred rhamnolipids homologues have been discovered mainly in* Pseudomonas* species even if several bacteria belonging to other genera like* Burkholderia* or* Acinetobacter* were also reported to produce rhamnolipids [27]. These amphiphilic biodegradable molecules have been reported to be useful as biological control agents due to their intrinsic wide-ranging antimicrobial potency [28]. For example, the mixture (six homologues) extracted from the* P. aeruginosa* LBI strains displayed a high activity against many bacterial strains like* Enterobacter aerogenes *and* Proteus mirabilis *with MIC between 4 and 8 *μ*g/ml [29]. Also, various bacterial species were sensitive to the* P. aeruginosa* 47T2 mixture (up to 11 homologues),* Enterobacter aerogenes* being by far the most sensitive one (MIC of 4 *μ*g/ml) [30]. To date, except for surfactin which was already described for its anti-*Legionella* activity [25], neither lipopeptide nor rhamnolipid mixture was reported to be active against bacteria of the* Legionella *genus.

The aim of our study was to discover and characterize natural anti-*Legionella *compounds produced by* Pseudomonas* strains. Therefore, a bacterial collection with both clinical and environmental strains was built and screened to find* Pseudomonas* sp. with the capacity to inhibit the growth of* L. pneumophila*. Biomolecules responsible for this antagonistic activity were then purified by RP-HPLC and their chemical structures were elucidated by LC-MS-MS. Antimicrobial activities were determined against selected Gram-positive and Gram-negative indicator strains including many* Legionella* species.

## 2. Materials and Methods

### 2.1. Bacterial Strains

*Pseudomonas* strains used in this study are listed in Table 1 while other bacterial strains are listed in Tables 5 and 6.* Pseudomonas* strains were routinely cultured at 28°C for nonaeruginosa strains or 37°C for* aeruginosa *strains either on LB agar plates or in LB broth under shaking (180 rpm). LB was composed of 5 g/l Yeast extract, 10g/l Tryptone and 5 g/l NaCl.* Legionella* strains were cultured at 37°C either on buffered charcoal yeast extract (BCYE) agar plates or in buffered yeast extract (BYE) liquid medium under shaking (150 rpm). BYE was composed of 5 g/l N-(2-Acetamido)-2-aminoethanesulfonic acid, 10 g/l Yeast extract and pH 6.9. BCYE was made from BYE by adding 2 g/l Activated charcoal and 15 g/l Agar.* L. pneumophila* Lens CIP 108286 [31] was used as the main target for anti-*Legionella* activity assays. Other bacteria were grown either on Brain Heart Infusion (BHI; Fisher Scientific, Illkirch, France) agar plates or broth, at 30°C or 37°C, depending on the tested strain.

### 2.2. Culture Conditions and Reagents

Rhamnolipids production was achieved by cultivating* Pseudomonas* strains at 30°C for 96 h under shaking (180 rpm) in a mineral salt medium (MSM) with mannitol (20 g.l^−1^) as the only carbon source [41]. For lipopeptides production,* Pseudomonas* strains were grown at 17°C for 96 h under shaking (180 rpm) in MSM medium supplemented with glucose (20 g.l^−1^).

A commercial solution of rhamnolipids was purchased from Sigma-Aldrich (Reference R95DD; Sigma-Aldrich, St. Louis, MO, USA). Rhamnolipids were dissolved in a water/acetonitrile (ACN) (65:35, v/v) mixture with 4 mM ammonium acetate at a final concentration of 5 mg.ml^−1^. Stock solutions were stored at 4°C and freshly diluted in sterile distilled water prior to each experiment. All other reagents were purchased from Sigma-Aldrich (Saint-Louis, MO, USA) unless stated otherwise.

### 2.3. In Vitro Antibacterial Assays

#### 2.3.1. Spot on Lawn Assay

The target* strain*,* L. pneumophila* Lens, was spread (100 *μ*l at OD_600_ = 0.1) onto a BCYE agar plate. Then, 10 *μ*l of overnight cultures of each* Pseudomonas *strain was spotted onto the surface of the agar plate before incubation for 96 h at 28°C or 37°C. An inhibition area, around the producing strain, revealed the antibacterial activity.

#### 2.3.2. Well Diffusion Assay

A* L. pneumophila* Lens suspension (100 *μ*l at OD_600_ = 0.1) was spread onto a BCYE agar plate. Wells were punched into the agar and filled with 100 *μ*l of 15 times concentrated supernatants of active* Pseudomonas* strains. Plates were then incubated 96 h at 37°C. Antibacterial activity was revealed by a zone of inhibition around the well.

#### 2.3.3. Determination of Minimum Inhibitory Concentrations

Minimum inhibitory concentrations (MICs) of biosurfactants towards various bacterial strains were measured according to the dilution method detailed elsewhere [42]. MIC was defined as the lowest concentration of biosurfactant required to totally inhibit the growth of a selected strain after a chosen incubation period (24 h or 96 h), depending on the tested strain.

### 2.4. Detection of Biosurfactants

#### 2.4.1. Drop Collapse Test

A drop of 50 *μ*l of each active* Pseudomonas* strain supernatant, containing 2.5 *μ*l of a 20 mg. ml^−1^ methylene blue solution, was placed on a piece of parafilm. Drops containing biosurfactants collapse, whereas nonsurfactant-containing drops remain stable. In this study, concentrated LB broth (15X) was used as a negative control while surfactin produced by* Bacillus subtilis* LMG 28342 [25] was used as a positive control.

#### 2.4.2. Amino Acids and Sugars Detection

The peptide moiety of lipopeptides was detected using a 0.25% ninhydrin solution (prepared in acetone and acetic acid). A sample volume of 10 *μ*l was spotted onto a silica plate (Saint-Louis, MO, USA). The plate was then sprayed with ninhydrin and heated at 105°C until development of a purple color. Surfactin produced by* Bacillus subtilis* LMG 28342 [25] was used as a positive control. The sugar moiety of rhamnolipids was detected using a 0.15% anthrone solution. A sample volume of 10 *μ*l was spotted onto a silica plate and the plate was then sprayed with anthrone. The presence of sugars was revealed by the development of a blue color. Glucose and a commercial solution of rhamnolipids were both used as positive controls.

### 2.5. Extraction of Biosurfactants

#### 2.5.1. Lipopeptides

Bacteria were removed from MSM culture medium by centrifugation (10,000 x g, 30 min, 4°C) and the resulting supernatant was sterilized by filtration through a 0.22 *μ*m syringe filter (Sarstedt AG & Co. KG, Germany). Then, the cell-free supernatant was extracted three times with ethyl acetate (1:1, v/v). The collected organic fractions were evaporated under vacuum, and the residue was dissolved in 5 ml of H_2_O/ACN (3:2, v/v). The resulting solution was termed “Lipopeptides raw extract”.

#### 2.5.2. Rhamnolipid

Bacteria were removed from MSM culture medium by centrifugation (10,000 x g, 30 min, 4°C) and the resulting supernatant was sterilized by filtration through a 0.22 *μ*m syringe filter (Sarstedt AG & Co. KG, Germany). The supernatant pH was then adjusted to 3 (using 1M HCl). The resulting acidified cell-free supernatant was extracted three times with ethyl acetate (1:1, v/v) and the collected organic fractions were evaporated under vacuum. The crude extract was then dissolved in 5 ml of H_2_O/ACN (3:2, v/v) and the solution was named “Rhamnolipids raw extract”.

### 2.6. Purification of Biosurfactants

#### 2.6.1. Lipopeptides

The “Lipopeptides raw extracts” were diluted in a H_2_O/ACN (1:1, v/v) mixture with 0.2% formic acid and separated by reverse-phase HPLC. Separation was conducted on a Chromolith® SpeedROD RP-18e reverse-phase HPLC column (4.6 x 50 mm) (Merck Millipore, Billerica, MA, USA) with a Dionex P680 HPLC pump, fitted with a Dionex UltiMate 3000 detector. Elution was monitored at 205 nm, 214 nm and 280 nm. Separation was carried out using a H_2_O/ACN/formic acid 0.2% (v/v) solvent system. After an initial 2 min wash with 60% ACN, elution was achieved in 23 min at a flow rate of 0.8 ml min^−1^ with an 18 min linear gradient from 60 to 100% ACN, followed by a 5 min wash with 100% ACN. All the collected fractions were lyophilized and stored at -20°C for further studies.

#### 2.6.2. Rhamnolipids

The “Rhamnolipids raw extracts” were diluted in a H_2_O/ACN (1:1, v/v) mixture with 4 mM ammonium acetate and separated by reverse-phase HPLC similarly as described above for lipopeptides. Separation was carried out using a H_2_O/ACN/4mM ammonium acetate solvent system. After an initial 4 min wash with 35% ACN, elution was achieved in 31 min at a flow rate of 0.4 ml min^−1^ with a 5 min linear gradient from 35% to 50% ACN, 50% ACN for 6 min, followed by a linear 20 min gradient from 50% to 90% ACN. Fractions were collected every minute, lyophilized and stored at -20°C for further studies.

### 2.7. Mass Spectrometry Analyses of Biosurfactants

The molecular masses of biosurfactants were determined by electrospray ionization mass spectrometry (ESI-MS) with a Xevo Q-TOF (Waters, Milford, MA, USA) mass spectrometer. Samples were suspended in 50% ACN/0.2% formic acid (v/v). LC-MS mass spectra were performed, in positive mode for lipopeptides and negative mode for rhamnolipids, with a cone voltage ramping from 20 to 40 V. The spray voltage was set to 3.0 kV, the source temperature to 120°C and the desolvation temperature to 450°C. The LC separation was conducted using the same column and gradient as for HPLC analyses indicated previously except for the flow rate which was reduced to 0.5 ml min^−1^. LC-MS/MS mass spectra were performed in the MS^E^ mode (Waters, Milford, MA, USA). Briefly, in the MS^E^ mode, mass spectrometric scans alternate all along the experiment between low (10 V) and high (ramping from 30 to 60 V) fragmentation energy delivering for the same LC separation two chromatograms corresponding to MS and MS/MS analyses, respectively.

## 3. Results

### 3.1. Screening of a Pseudomonas Collection

Twenty-one* Pseudomonas* sp. strains of environmental or clinical origin and representing eight different species were screened for antagonistic activity against* L. pneumophila* Lens using a spot on lawn assay. As presented in Table 1, an inhibition zone was observed around all colonies of* P. aeruginosa *(3/3),* P. fluorescens* (5/5), and, with a smaller diameter,* P. syringae* (2/2).* P. otitidis* 4014 and* P*. sp. DSS73 also displayed a large zone of inhibition. Surprisingly, only one of the 4* P. putida* strains tested appeared to be active against* L. pneumophila* Lens. Therefore, these strains inhibited* L. pneumophila* Lens growth via the production of, at least, one diffusible active compound. On the contrary, no zone of inhibition was observed around colonies of all tested* P. fulva* strains (3/3) as well as* P. cepacia* 4512 and* P. libanensis* 4000. These strains did not secrete any anti-*Legionella* compound or in too low concentration to be detected.

In order to demonstrate that antimicrobial compounds were effectively secreted by all the active strains, their culture supernatants were also tested against* L. pneumophila *Lens by well diffusion assay on BCYE plates (Table 1). Crude supernatants did not show any antibacterial activity but after being concentrated 15 times, an anti-*Legionella* activity, related to a zone of inhibition around the well containing samples, was observed. Anti-*Legionella* compounds were indeed secreted by active bacteria cultivated in broth medium, but at quite low amounts.

### 3.2. Chemical Nature of Active Compounds

Different members of the genus* Pseudomonas* are known to produce biosurfactants with antimicrobial activities [15, 28]. To check if the anti-*Legionella *activity could be attributed to biosurfactants, their presence in the culture supernatant was determined by the drop collapse test. This qualitative test is indicative of the surface-active and wetting activities [43] and it represents an indirect measurement of surface activity of a biosurfactant. Interestingly, the drops of culture supernatant for all inactive strains remained stable, whereas the culture supernatant of all active strains induced a drop collapse (Table 2). These results indicate that the anti-*Legionella* compounds secreted by* Pseudomonas* strains were correlated to the presence of biosurfactants. Therefore, putative biosurfactants were extracted from culture supernatants using ethyl acetate as previously described [41, 44]. The subsequent extracts were found active against* L. pneumophila* when tested using well diffusion assay (Table 1). On the contrary, similar extracts obtained from inactive strains did not display any activity. Moreover, all aqueous extracts that correspond to the remaining cell-free supernatants after extraction with ethyl acetate were found inactive too.

The extracts obtained from the three* P. aeruginosa *strains and* P. otitidis* 4014 were found positively colored by anthrone, but not ninhydrin, revealing the presence of glucidic compounds whereas all the other active extracts were only colored by ninhydrin (Table 2). These results indicated that tested* P. aeruginosa* strains could produce rhamnolipids as well as* P. otitidis* 4014. Other active strains were found to produce biosurfactants containing peptidic moieties, probably lipopeptides. Finally, inactive strains did not produce any glucidic or peptidic biosurfactants as indicated by the lack of coloration (Table 2).

### 3.3. Purification and Identification of Lipopeptides

*P. fluorescens* (MAFD21c, DSS73, MFAO2, PfA7b, and MFAH4a) and* P. putida* MFAF88 were grown in MSM medium [44] and lipopeptides were extracted with ethyl acetate. For each strain, RP-HPLC chromatograms of ethyl acetate extracts displayed at least two peaks (data not shown) which could correspond to lipopeptides, according to Janek and coworkers [44]. In parallel, extracts were analyzed by LC-MS and LC-MS/MS in order to characterize active molecules. Results of these analyses are summarized in Table 3. All of the active fractions were found to contain a molecule displaying a molecular mass already described in the literature (Table 3) except for the* P. *sp. DSS73 fraction with a retention time of 11.9 min. The latter one molecular mass, which was named Amphisin-like, is reduced by 14 Da as compared to Amphisin and could correspond to the replacement of a leucine residue by a valine in the peptidic part of the molecule. The other lipopeptides produced by this strain correspond to Amphisin and Tensin which were also found in the* P. fluorescens* MFAO2 extract (Table 3).* P. putida* MFAF88 lipopeptides were identified as Putisolvin I and II. Massetolide E and Viscosin were identified in the* P. fluorescens* PfA7b extract. Finally,* P. fluorescens* MHA4a and MFAD21c were both found to produce PPZPM-1a and PPZPM-2a. Finally, full 1:1 identification is likely for the various lipopeptides, but must await further confirmation through chemical or genomic sequence analysis (Supplementary Table S1).

To estimate the proportion between lipopeptides contained in each extract, the relative quantity for each molecule was measured (Table 3). For some strains, in our conditions, lipopeptides were produced in similar amounts (*P. fluorescens* PfA7b and* P. fluorescens* MFAH4a) whereas for most of strains (*P. fluorescens* MFAO2, MFAD21c,* P*. sp. DSS73, and* P. putida* MFAF88) proportions of lipopeptides produced were really dissimilar. Thus, proportions of Amphisin, Amphisin-like and Tensin produced by the strain DSS73 were 78.4%, 18.2% and 3.4%, respectively, whereas for* P. fluorescens *MFAO2, the biosurfactant mixture was composed of 6.1% of Amphisin and 93.9% of Tensin.

### 3.4. Purification and Identification of Rhamnolipids

**Figure 1:**
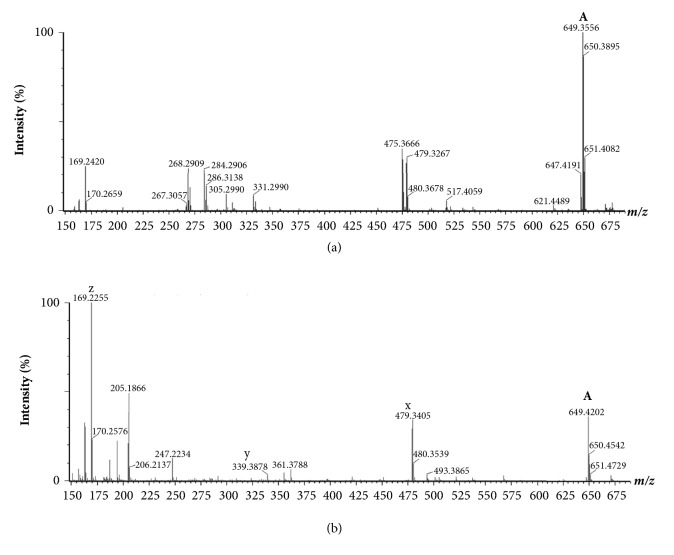
MS (a) and MS/MS (b) spectra of the RP-HPLC fraction eluted at 19 min from* P. aeruginosa* CHA extract.

**Figure 2:**
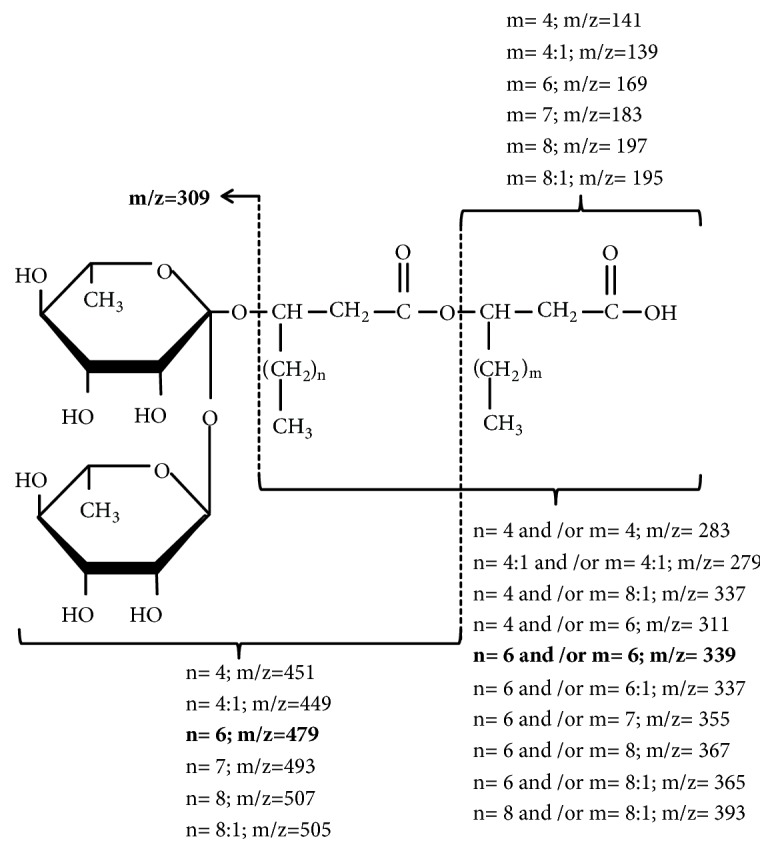
Fragmentation of di-rhamnolipids observed in MS/MS analysis. The* m/z* of the fragments as a function of the structures of fatty acids is indicated.

To characterize the chemical structure of rhamnolipids produced by* P. aeruginosa* strains (8H, CHA and UCBPP-PA14) and* P. otitidis *4014, bacteria were cultivated in MSM broth containing mannitol as the only carbon source [41]. Rhamnolipids extracted with ethyl acetate from culture supernatants were then separated using RP-HPLC. Because rhamnolipids have no UV-absorptive properties, fractions were blind collected every minute during the elution and then tested against* L. pneumophila*. In parallel, extracts were analyzed by LC-MS and LC-MS/MS in order to characterize active molecules. All fractions, which were found active against* Legionella*, contained rhamnolipids with at least two congeners. Rhamnolipids consist of one or two units of rhamnose linked to one or two hydroxylated fatty acid with C8 to C12 chains, which could be saturated or not. The molecular masses of pseudomolecular ions and characteristic fragments observed, respectively, in MS and MS/MS spectra are listed in Table 4. Several types of rhamnolipids congeners of molecular masses in the range m/z 473-703, depending on their number of rhamnose residues and the length of their fatty acid chains, were observed. Structural characterization was achieved using MS/MS spectra by detection of characteristic fragments [29] and an example is detailed on Figures 1 and 2. The m/z of the pseudomolecular ion [M-H]^−^ of component A is 649 Da (Figure 1(a)). Thus, the parent ion at m/z 649 was fragmented by MS/MS and showed daughter ions at m/z 479 (x), 309, 339 (y), and 169 (z) (Figure 1(b)). Fragment at m/z 309 is characteristic of di-rhamnolipids. Indeed, it corresponds to the di-rhamnosyl residue (Figure 2). The fragment at m/z 339 corresponds to the lipid moiety composed of two hydroxylated fatty acids containing ten carbons (C10). The fragment at m/z 479 results from the rupture of the ester bond between the two fatty acids. This fragment is characteristic of di-rhamnolipids carrying a C10 hydroxylated fatty acid directly linked to the carbohydrate part of the molecule. Taken together, the component A is a di-rhamnolipid carrying two C10 hydroxylated fatty acids (Rha-Rha-C_10_ -C_10_) (Figure 2).

Up to 29 rhamnolipids homologues containing one or two rhamnose residues linked to one or two hydroxylated fatty acids were identified in* Pseudomonas* culture supernatants (Table 4).* P. aeruginosa* PA14 produced the highest number of homologues (25) and the lowest was found for* P. aeruginosa* 8H (11). Only 7 rhamnolipid homologues (Rha-C_10_ C_8_; Rha-C_10_ C_10_; Rha-Rha-C_10_ C_8_; Rha-Rha-C_10_ C_10_; Rha-Rha-C_10_ C_12:1_; Rha-Rha-C_10_ C_12_; Rha-Rha-C_10_) were produced by the 3 strains. Interestingly,* P. aeruginosa* 8H secreted only di-rhamnosyl carrying two fatty acids species.

Finally, the orcinol reaction revealed that extracts contained rhamnose amounts of 79.95 ± 16.61 mg/ml for* P. aeruginosa *8H, 66.84 ± 3.43 mg/ml for* P. aeruginosa *CHA, 10.99 ± 4.18 mg/ml for* P. aeruginosa* UCBPP-PA14 and 4.07 ± 1.56 mg/ml for* P. otitidis* 4014. It has to be noted that the rhamnolipid content of* P. aeruginosa *8H extract is overestimated because it contains only di-rhamnosyl rhamnolipids contrarily to the other strains extracts.

### 3.5. Anti-Legionella Activity of Biosurfactants

#### 3.5.1. Lipopeptides

Firstly, HPLC fractions corresponding to identified lipopeptides were collected, concentrated and tested against* L. pneumophila*. All fractions were found active except those corresponding to the molecules with the lowest proportions, Putisolvin II (23.4%), Amphisin-like (18.2%), Tensin in DSS73 extract (3.4%) and Amphisin in MFAO2 extract (6.1%) (Table 3). Because Amphisin and Tensin were found active when obtained in larger amounts from other extracts, we supposed that Tensin from the strain DSS73, Putisolvin II and Amphisin-like appeared inactive because of their low concentration. It has to be noted that it is impossible to quantify the amounts of lipopeptides using classical colorimetric methods. To confirm this proposal, fractions corresponding to each of these two molecules were obtained from more than twenty HPLC runs and pooled before being concentrated. In both cases, fractions were found active against* L. pneumophila*. In conclusion, all the lipopeptides produced by* Pseudomonas* species were found to be active against* L. pneumophila*.

Secondly, the antagonistic potency of the purified lipopeptide mixtures was determined against various bacterial strains previously used in antibacterial assays [25, 45, 46]. Because it was difficult to quantify the lipopeptide content of extracts, activities were expressed as a function of the first twofold dilution of the extract which totally inhibited the growth of the target bacteria (Table 5). All of the mixtures were more or less active against* Legionella *species. However, mixtures were not active against the other Gram-negative or Gram-positive bacteria tested so far. Thus, these results highly suggest a specific activity of lipopeptide mixtures against bacteria of the* Legionella* genus. The* P. fluorescens *MFAH4a extract seemed to be the less active one, with activities observed only for undiluted to four times diluted solutions. On the contrary,* P. fluorescens *PfA7b extract appeared to be the most antibacterial extract (Table 5).

#### 3.5.2. Rhamnolipids

MICs of the rhamnolipid mixtures produced by* Pseudomonas *strains cultured in MSM broth were determined against the same collection of bacterial strains used for lipopeptides. Results are given in Table 6. Extracts were found highly active against all the* Legionella* sp. tested with low MIC values between 0.027 and 25 *μ*g/ml. The mixture produced by* P. aeruginosa* 8H was the most effective against* Legionella* sp, with the lowest MIC values. On the opposite, the extract originating from* P. aeruginosa* CHA culture supernatant and commercial rhamnolipid mixture displayed 10 to 100-fold higher MICs.

Interestingly, rhamnolipid mixtures were found less or not active against other tested bacteria.* B. subtilis* appeared sensitive to the rhamnolipid extracts but not to commercial mixture. The* P. aeruginosa* 8H extract displayed activity against most of the other bacteria tested but not* S. aureus*. In contrast*, S. aureus* was found sensitive to* P. aeruginosa* CHA (52.5 *μ*g/ml) extracts. Finally, the* P. aeruginosa* 8H extract appears about 2 to 10 times more active than the one from* P. aeruginosa* PA14 and displays an anti-*Legionella* activity from 5 to 50 times higher than both* P. aeruginosa* CHA and the commercial mixture. Taken together, these results indicate that* Legionella* sp. are particularly sensitive to the rhamnolipid mixtures produced by* Pseudomonas* sp.

In order to evaluate the activity of various homologues of rhamnolipids, HPLC fractions obtained from the four producing strains were tested against* L. pneumophila* and then their content was analyzed by LC-MS in order to identify all the rhamnolipid species (Data not shown). All fractions were found more or less active against* Legionella* according to their amount of rhamnolipids, estimated from their peaks area in LC-MS chromatograms (data not shown). However, all these fractions were found to contain at least two rhamnolipid homologues. Consequently, even if many of these molecules are undoubtedly active against* Legionella*, it is not possible to affirm which one is effectively active against* Legionella*.

## 4. Discussion

Artificial water settings provide suitable conditions for growth and multiplication of waterborne pathogens including* L. pneumophila*. In those nutrient-poor environments,* L. pneumophila* is able to interact positively with other microorganisms to obtain the nutrients it requires to survive [9, 47]. Although much work has been conducted on the stimulation of* Legionella* growth by other microorganisms, little work has been done on the negative interactions that occur between* Legionella *bacteria and other microorganisms in man-made water systems. Compilation of latest findings shows that many bacterial genera isolated from drinking water pipes were able to inhibit the growth of* Legionella* species [13, 48–50]. While active isolated strains were taxonomically diverse, bacteria belonging to the* Pseudomonas* genus were always found or tested. Another constant of those studies is the lack of molecular identification of active compounds that have been thought to be bacteriocins or bacteriocin-like substances [13, 48].

To further investigate the chemical nature of those anti-*Legionella *compounds, a* Pseudomonas *sp. collection, comprising both environmental and clinical strains, was defined and screened. Among the 21 tested strains, 14 were active against* L. pneumophila* (66.7%). This result is in good agreement with the data published by Guerrieri and coworkers as they found 72% of active* Pseudomonas* strains in their collection [13]. The anti-*Legionella* compounds secreted by* Pseudomonas* strains were then correlated to the presence of biosurfactants, as* Pseudomonas* members are well known to produce many biosurfactants with antimicrobial activities [15, 28].

Lipopeptides constitute a specific class of microbial secondary metabolites produced by a wide range of microorganisms. Moreover, those produced by* Bacillus* and* Pseudomonas *species are the most studied by far [17]. Here, all active fractions purified from five* P. fluorescens* and one* P. putida* ethyl acetate extracts contained various already known lipopeptides. According to the literature, only the molecule with a molecular mass of 1383 Da is original. Thus, this lipopeptide was named Amphisin-like as its molecular mass is 14 Da lower than the molecular mass of Amphisin [39]. We also found lipopeptides belonging to the orfamide group named PPZPMs, a group of CLPs thought to be the missing link between the viscosin and Amphisin groups due to the number of amino acids forming the cyclic moiety [22, 38]. Lipopeptides are mainly characterized by highly structural diversity and are considered as multifunctional microbial tools. Indeed, they exhibit a very wide range of biological activities including lytic and growth-inhibitory activities against a broad range of microorganisms [14]. In particular, many authors have reported antibacterial activities (for review see [17]). Usually, Gram-negative bacteria are poorly inhibited by lipopeptides whereas Gram-positive bacteria appear more susceptible [15, 17, 19]. To date, only one lipopeptide was reported to be active against* Legionella* species [25]. It corresponds to surfactin, a well characterized lipopeptide produced by* Bacillus* species. However, all the lipopeptides tested were found active against* Legionella* species. It is the first time, to our knowledge, that* Pseudomonas* biosurfactants were shown to be active against* Legionella. *Interestingly, other Gram-negative and Gram-positive bacteria tested were insensitive to those compounds used at a similar concentration. In many studies, when available, MIC values of lipopeptides against bacteria ranged from less than 10 *μ*g/ml (massetolide A and viscosin against* M. tuberculosis*) to around 1 mg/L (milkisin against* S. enterica*) [37, 51]. Thus, MIC determined in this study were in a similar concentration range. Nevertheless,* Legionella* species appeared more sensitive to surfactin than to* Pseudomonas* lipopeptides as MIC values were lower (1-4 *μ*g/ml) [25]. Interestingly, Loiseau and coworkers showed that nonlegionella bacterial strains tested were resistant to surfactin, even at the highest tested concentration (265 *μ*g/mL). Taken together, those data highlight a very specific sensitivity of* Legionella* bacteria to lipopeptides.

Interestingly, rhamnolipids extracts were active against all the* Legionella* tested, whatever their species. The percentage of di-rhamno-di-lipid in each mixture was higher than those of monorhamno-di-lipid and di-rhamno-monolipid. This result is in good agreement with previous observation of Arino and coworkers [52]. Interestingly, the* P. aeruginosa* 8H extract, which is the more active, contained the lower number of rhamnolipid congeners as compared to other extracts. It could be related to the higher concentration of each rhamnolipid molecule in the extract. Indeed, in all cases, the rhamnolipid content was evaluated as a function of its rhamnose content. Thus, the mean concentration of each congener is higher for* P. aeruginosa* 8H which contained 11 different rhamnolipids than those of* P. aeruginosa *CHA (16 congeners), PA14 (25 congeners) and* P. otitidis* 4014 (13 congeners). However, this cannot explain the higher activity of* P. aeruginosa* PA14 as compared to* P. aeruginosa* CHA. The other main difference of* P. aeruginosa* 8H extract as compared to the others corresponds to its content restricted to di-rhamnosyl species which could then be related to its higher anti-*Legionella* activity. Moreover, this* P. aeruginosa* 8H extract was found to be the most active towards no-*Legionella* strains except* S. aureus* and* B. subtilis* for which the* P. aeruginosa* PA14 and* P. otitidis* 4014 extracts were found the most active. On the other hand, the insensitivity of* S. aureus* to the* P. aeruginosa* 8H extract could be related to its particular content restricted to di-rhamnosyl species. Indeed, the comparison of the rhamnolipids congeners produced by* P. aeruginosa* PA14 (this study), CHA (this study), AT10 [53], LBI [29] and RL 47T2 [30] did not reveal the presence of a specific anti-*Staphylococcus aureus* compound. However, all these extracts contained monorhamnosyl species which could be postulated to exert this specific activity. Strikingly,* Legionella* species were shown to be highly sensitive to rhamnolipid mixtures as MIC values were quite low (0.03-19.5 *μ*g/ml) while throughout the literature, many sensitive bacterial species displayed higher MIC values [27, 28]. Does* Legionella* bacteria possess some specificity that could explain this high sensitivity? As these compounds are membrane active, maybe a part of the answer is hidden in the composition of the cell envelope. Indeed,* Legionella* are also highly sensitive to detergents (SDS, Tween 80, Triton X-100…) or detergent-like molecules such as antimicrobial peptides [25, 45, 54, 55]. Thus, the phospholipid composition of* Legionella *cell envelope as well as the membrane thickness, the fluidity, the presence of phospholipids clusters and even composition of the lipopolysaccharide could be key parameters involved in* Legionella* sensitivity towards membrane-active compounds [56].

## 5. Conclusions

In this study, we showed, for the first time, that biological challengers present in the microenvironment of* Legionella* such as* Pseudomonas* bacteria exhibit antagonistic activity because of the production of various biosurfactants species. These compounds are known to be multifunctional biomolecules with many depicted potential biotechnological applications including their use as antimicrobials [57]. Thus, the wide sensitivity of* Legionella* species to rhamnolipids and lipopeptides make biosurfactants promising tool for their biological control in water treatment industry although experimental data are needed to evaluate how effective biosurfactants would be in real conditions.

## Figures and Tables

**Table 1:** Anti-*Legionella  pneumophila* activity of *Pseudomonas *strains.

***Pseudomonas strains***	**Anti-*Legionella*** * * **activity**_ _^**a**^		**Reference/Source**_ _^**b**^
	Colony	Concentrated supernatant (15X)	
*P. aeruginosa* 8H	+++	+++	EBI collection
*P. aeruginosa* CHA	+++	+++	[32]
*P. aeruginosa *UCBPP-PA14	+++	+++	[33]
*P. cepacia* 4512	-	-	EBI collection
*P. *sp DSS73	+++	++	[34]
*P. fluorescens* MFAD21c	+++	+	[35]
*P. fluorescens* MFAH4a	+++	+	[35]
*P. fluorescens* MFAO2	+++	++	[35]
*P. fluorescens *MFE01	+++	+	[36]
*P. fluorescens* PfA7b	+++	+	LMSM collection
*P. fulva* 1324	-	-	EBI collection
*P. fulva* B6	-	-	EBI collection
*P. fulva* B8	-	-	EBI collection
*P. libanensis* 4000	-	-	EBI collection
*P. otitidis* 4014	+++	+	EBI collection
*P. putida* 1243	-	-	EBI collection
*P. putida* 1312	-	-	EBI collection
*P. putida* MFAF88	++	+	[35]
*P. putida* MFAK14	-	-	[35]
*P. syringae* MFAA66a	+	-	[35]
*P. syringae* MFAE88	+	-	[35]

The anti-*Legionella* activity of the concentrated supernatant for each strain grown in LB medium is the mean of three independent experiments.

^a^-: no activity.

+: zone of inhibition with a diameter < 4 mm.

++: zone of inhibition with a diameter between 4 and 8 mm.

+++: zone of inhibition with a diameter > 8 mm.

^b^EBI: Laboratoire Ecologie & Biologie des Interactions, UMR CNRS 7267, Université de Poitiers; LMSM: Laboratoire de Microbiologie Signaux et Microenvironnement, EA4312, Université de Rouen.

**Table 2:** Determination of the chemical nature of active compounds produced by *Pseudomonas* strains.

***Pseudomonas strains***	**Drop collapse assay**	**Ethyl acetate extract activity**	**Coloration by anthrone**	**Coloration by ninhydrin**
*P. aeruginosa* 8H	+	+	+	-
*P. aeruginosa* CHA	+	+	+	*-*
*P. aeruginosa *UCBPP-PA14	+	+	+	-
*P. cepacia* 4512	-	-	-	-
*P. sp *DSS73	+	+	-	+
*P. fluorescens* MFAD21c	+	+	-	+
*P. fluorescens* MFAH4a	+	+	-	+
*P. fluorescens* MFAO2	+	+	-	+
*P. fluorescens *MFE01	+	+	-	+
*P. fluorescens* PfA7b	+	+	-	+
*P. fulva* 1324	-	-	-	-
*P. fulva* B6	-	-	-	-
*P. fulva* B8	-	-	-	-
*P. libanensis* 4000	-	-	-	-
*P. otitidis* 4014	+	+	+	-
*P. putida* 1243	-	-	-	-
*P. putida* 1312	-	-	-	-
*P. putida* MFAF88	+	+	-	+
*P. putida* MFAK14	-	-	-	-
*P. syringae* MFAA66a	+	+	-	+
*P. syringae* MFAE88	+	+	-	+

+: three independent assays give a positive result.

-: three independent assays give a negative result.

**Table 3:** Pseudomolecular ions and main product ions obtained in positive mode from ESI-MSn (n=1, 2) analyses of purified active HPLC fractions from ethyl acetate extract of *Pseudomonas *sp. cell-free supernatants.

**Producing strain**	**Name**	**[M+H]** ^**+**^	**Main product ions in MS-MS (m/z)**	**R**_**t**_ ** [min]**	**Relative area (**%**)**	**Anti-*L. pneumophila* activity**	**Reference**
*P. putida* MFAF88	Putisolvin I	1380.7	454; 567; 863; 927; 1040; 1169	14.7	76.6	+	[20]
	Putisolvin II	1394.7	1054; 1183	15.7	23.4	-	

*P. fluorescens* PfA7b	Massetolide E	1112.6	496; 595; 829; 700	13.9	54.8	+	[37]
	Viscosin/Massetolide F	1126.6	496; 595; 714; 843	14.8	45.2	+	

*P. fluorescens* MFAD21c	PPZPM-1a	1253.8	284; 496; 609; 970; 1235	11.2	61.7	+	[38]
	PPZPM-2a	1239.8	284; 496; 595; 956; 1221	12.2	38.3	+	

*P. fluorescens* MFAH4a	PPZPM-1a	1253.8	284; 496; 609; 970; 1235	11.2	53.1	+	[38]
	PPZPM-2a	1239.8	284; 496; 595; 956; 1221	12.1	46.9	+	

*P. *sp DSS73	Amphisin	1395.6	482; 595; 997; 1112	12.5	78.4	+	[39, 40]
	Amphisin-like	1384.7	482; 595; 997; 1098	11.9	18.2	-	
	Tensin	1409.5	482; 595; 1011; 1026	13.2	3.4	-	

*P. fluorescens* MFAO2	Amphisin	1395.6	482; 595; 997; 1112	12.7	6.1	-	[39, 40]
	Tensin	1409.8	482; 595; 1011; 1026	13.2	93.9	+	

**Table 4:** Chemical composition of rhamnolipid mixtures produced by *Pseudomonas* strains.

**Rhamnolipid structure**	[**M**-**H**]^−^ **(*m/z*)**	**Ion fragments** **(*m/z*)**	***Pseudomonas *strain**			
			**PA14**	**8H**	**CHA**	**4014**

**Mono-rhamno-di-lipid**						

**Rha-C8-C8**	447	Nd	**+**	-	-	-

**Rha-C10-C8:1**	473	327	-	-	-	**+**

**Rha-C8:1-C10**	473	333	-	-	-	**+**

**Rha-C10-C8**	475	333	+	-	+	+

**Rha-C8-C10**	475	305	+	-	+	+

**Rha-C10-C10**	503	339, 333, 169	**+**	-	**+**	**+**

**Rha-C10-C12:1**	529	333, 195	**+**	-	**+**	-

**Rha-C12:1-C10**	529	359, 169	**+**	-	**+**	-

**Rha-C10-C12**	531	333	**+**	-	-	-

**Rha-C12-C10**	531	361	**+**	-	-	-

**Di-rhamno-mono-lipid**						

**Rha-Rha-C8**	451	Nd	**+**	-	**+**	-

**Rha-Rha-C10**	479	Nd	**+**	-	**+**	-

**Rha-Rha-C12:2**	503	Nd	**+**	-	**+**	-

**Di-rhamno-di-lipid**						

**Rha-Rha-C8:1-C8:1**	589	449, 309, 279, 140	-	-	-	**+**

**Rha-Rha-C8-C8**	593	451, 142	**+**	**+**	**+**	-

**Rha-Rha-C10-C8**	621	479, 142	**+**	**+**	**+**	**+**

**Rha-Rha-C8-C10**	621	451, 169	**+**	**+**	**+**	**+**

**Rha-Rha-C10-C10:1**	647	479, 337, 309, 167	-	**+**	-	-

**Rha-Rha-C8-C12:1**	647	451	**+**	-	-	-

**Rha-Rha-C12:1-C8**	647	505	**+**	-	-	-

**Rha-Rha-C10-C10**	649	479, 339, 309, 169	**+**	**+**	**+**	**+**

**Rha-Rha-C11-C10**	665	494, 309, 169	**+**	**+**	-	-

**Rha-Rha-C10-C11**	665	479, 309, 184	**+**	**+**	-	-

**Rha-Rha-C10-C12:1**	675	479, 365, 309, 195	**+**	**+**	**+**	**+**

**Rha-Rha-C12:1-C10**	675	505, 365, 309, 169	**+**	**+**	**+**	**+**

**Rha-Rha-C10-C12**	677	479, 367, 309, 197	**+**	**+**	**+**	**+**

**Rha-Rha-C12-C10**	677	507, 367, 309, 169	**+**	**+**	**+**	**+**

**Rha-Rha-C12-C12:1**	703	507, 311	**+**	-	-	-

**Rha-Rha-C12:1-C12**	703	505, 311	**+**	-	-	-

**+: **presence of the rhamnolipid in the extract.

**-: **absence of the rhamnolipid in the extract.

**Nd: **not detected.

**Table 5:** Antibacterial spectrum of lipopeptide mixtures produced by *Pseudomonas* strains.

		**MIC of lipopeptide mixtures (mg/ml)**					
		DSS73	MFAD21c	MFAF88	MFAH4a	MFAO2	PfA7b
Gram positive	*B. subtilis *LMG 28342	-	-	-	-	-	-
	*E. faecalis *V583	-	-	-	-	-	-
	*L. ivanovii* Li4pVS2	-	-	-	-	-	-
	*L. monocytogenes *EGDe ATCC BAA-679	-	-	-	-	-	-

Gram negative	*A. hydrophila *LMG 2844	-	-	-	-	-	-
	*E. coli* LMG 2092	-	-	-	-	-	-
	*F. breve *LMG 4011	-	-	-	-	-	-
	*K. pneumoniae *050283	-	-	-	-	-	-
	*P. aeruginosa *LMG 1242	-	-	-	-	-	-
	*P. aeruginosa *UCBPP-PA14	-	-	-	-	-	-
	*S. enterica *J18	-	-	-	-	-	-
	*L. bozemanii* ATCC 33217	0.09	0.14	0.61	0.19	0.38	0.20
	*L. dumoffii *ATCC 33279	0.09	0.14	0.30	0.19	0.38	0.20
	*L. feeleii* ATCC 35072	0.09	0.14	0.15	0.19	0.04	0.20
	*L. longbeachae* ATCC 33484	0.18	0.14	0.30	0.19	0.19	0.20
	*L. micdadei *ATCC 33218	0.18	0.27	0.30	0.19	0.19	0.20
	*L. pneumophila* ATCC 33155 (Sg 3)	0.18	0.27	0.61	0.38	0.38	0.40
	*L. pneumophila* ATCC 33215 (Sg 6)	0.36	0.27	0.61	0.38	0.38	0.40
	*L. pneumophila* ATCC 33216 (Sg5)	0.18	0.27	0.30	0.38	0.19	0.40
	*L. pneumophila* ATCC Baa74 130b	0.36	0.27	0.61	0.76	0.38	0.40
	*L. pneumophila* Corby (Sg1)	0.18	0.27	0.61	0.38	0.38	0.40
	*L. pneumophila* Lens CIP 108286 (Sg 1)	0.36	0.54	0.61	0.76	0.38	0.40

-: no growth of inhibition was detected even with undiluted lipopeptide mixture (0.72 mg/ml for the DSS73 mixture, 1.08 mg/ml for the MFAD21c mixture, 0.76 mg/ml for the MFAH4a mixture, 0.76 mg/ml for the MFAO2 mixture, 1.6 mg/ml for the PfA7b mixture, and 1.22 mg/ml for the MFAF88 mixture).

Bacteria (10^6^ CFU/ml) were incubated in BYE or BHI medium with two-fold dilutions of lipopeptide mixtures. Results correspond to the MIC after incubation for 24h or 96 h at 37°C depending on the tested strain and are the mean of three independent experiments.

Bacterial strains were obtained from various culture collections: ATCC: American Type Culture Collection, CIP: Collection Institut Pasteur, France, and LMG: BCCM/LMG Bacteria Collection, Ghent University, Belgium. The Corby strain was kindly provided by the Centre National de Référence des Légionelles (Lyon, France). Other strains were from the laboratory culture collection. Sg: serogroup.

**Table 6:** Antibacterial spectrum of rhamnolipid mixtures produced by *Pseudomonas *strains.

	**MIC of rhamnolipid mixtures (** **µ** **g/ml)**				
	8H	CHA	PA14	4014	Commercial mixture
*A. hydrophila *LMG 2844	-	-	-	-	-
*B. subtilis *AM1	41.2	80	26.3	100	-
*E. faecalis *V583	41.2	-	-	-	-
*E. coli* DH5Y	20.6	-	-	-	-
*F. breve *LMG 4011	82.5	-	-	-	-
*K. pneumoniae *050283	165	-	-	-	-
*L. bozemanii* ATCC 33217	0.2	6.2	1.6	5.2	7.8
*L. dumoffii *ATCC 33279	0.4	12.5	0.4	5.2	15.6
*L. feeleii* ATCC 35072	0.2	6.2	0.4	3.9	11.7
*L. longbeachae* ATCC 33484	0.4	12.5	3.3	7.9	13.1
*L. micdadei *ATCC 33218	0.03	3.1	3.3	3.9	15.6
*L. pneumophila* ATCC 33155 (Sg 3)	0.2	12.5	3.3	15.8	8.8
*L. pneumophila* ATCC 33215 (Sg 6)	0.2	6.2	0.4	7.9	3.9
*L. pneumophila* ATCC 33216 (Sg5)	0.05	25	1.6	7.9	19.5
*L. pneumophila* ATCC Baa74 130b	0.05	12.5	0.4	7.9	15.6
*L. pneumophila* Corby (Sg1)	0.2	12.5	3.3	5.2	11.7
*L. pneumophila* Lens CIP 108286 (Sg1)	0.4	12.5	1.6	10.5	11.7
*L. ivanovii* Li4pVS2	41.2	-	-	-	-
*L. monocytogenes EGDe *ATCC BAA-679	41.2	-	-	-	-
*P. aeruginosa *LMG 1242	-	-	-	-	-
*P. aeruginosa *PA14	82.5	-	-	-	-
*S. enterica *J18	82.5	-	-	-	-
*S. aureus *ATCC 29213	-	805	52.5	50	-

-: no growth of inhibition was detected even with undiluted rhamnolipid mixture (660 *µ*g/ml for the 8H mixture, 1610 *µ*g/ml for the CHA extract, 105 *µ*g/ml for the PA14 extract, 100 *µ*g/ml for the 4014 extract, and 250 *µ*g/ml for the commercial mixture).

Bacteria (10^6^ CFU/ml) were incubated in BYE or BHI medium with two-fold dilutions of rhamnolipid mixtures. Results correspond to the MIC after incubation for 24h or 96 h at 37°C depending on the tested strain and are the mean of three independent experiments.

Bacterial strains were obtained from various culture collections: ATCC: American Type Culture Collection, CIP: Collection Institut Pasteur, France, and LMG: BCCM/LMG Bacteria Collection, Ghent University, Belgium. The Corby strain was kindly provided by the Centre National de Référence des Légionelles (Lyon, France). Other strains were from the laboratory culture collection. Sg: serogroup.

## Data Availability

The data used to support the findings of this study are available from the corresponding author upon request.

## Supplementary Materials

Supplementary Materials Table S1: lipopeptide structures detected in active HPLC fractions from ethyl acetate extract of* Pseudomonas* sp. cell-free supernatants according to their pseudomolecular ion masses as described in the literature.
