# Supplementary material for: Highlighting the Potency of Biosurfactants Produced by Pseudomonas Strains as Anti-Legionella Agents
Source: Biomed Res Int. 2018 Oct 22;2018:8194368. doi: 10.1155/2018/8194368 (PMC6217892; doi:10.1155/2018/8194368)
Supplement: Supplementary Materials — Table S1: lipopeptide structures detected in active HPLC fractions from ethyl acetate extract of Pseudomonas sp. cell-free supernatants according to their pseudomolecular ion masses as described in the literature. [file 8194368.f1.doc]

TABLE S1: Lipopeptide structures detected in active HPLC fractions from ethyl acetate extract of *Pseudomonas* sp. cell-free supernatants according to their pseudo molecular ion masses as described in the literature.

| **Amino acids** | | | | | | | | | | | | | | | |
| --- | --- | --- | --- | --- | --- | --- | --- | --- | --- | --- | --- | --- | --- | --- | --- |
| **Name** | **Bound Fatty Acid** | **1** | **2** | **3** | **4** | **5** | **6** | **7** | **8** | **9** | **10** | **11** | **12** | **[M+H]^+^** | **Reference** |
| Putisolvin I | CH_3_(CH_2_)_4_-CO- | L | E | L | I | Q | S | V | I | S | L | V | S | 1380 | [20] |
| Putisolvin II | CH_3_(CH_2_)_4_-CO- | L | E | L | I | Q | S | V | I | S | L | L/I | S | 1394 |
| Massetolide E | CH_3_-(CH_2_)_6_-CHOH-CH_2_-CO- | L | E | T | V | L | S | L | S | V |  |  |  | 1112 | [44] |
| Massetolide F | CH_3_-(CH_2_)_6_-CHOH-CH_2_-CO- | L | E | T | V | L | S | L | S | L |  |  |  | 1126 |
| Viscosin | CH_3_-(CH_2_)_6_-CHOH-CH_2_-CO- | L | E | T | V | L | S | L | S | I |  |  |  | 1126 |
| PPZPM-1a | CH_3_-(CH_2_)_6_-CHOH-CH_2_-CO- | L | E | T | I | L | S | L | L | S | I |  |  | 1253 | [43] |
| PPZPM-2a | CH_3_-(CH_2_)_6_-CHOH-CH_2_-CO- | L | E | T | V | L | S | L | L | S | I |  |  | 1239 |
| Amphisin | CH_3_-(CH_2_)_6_-CHOH-CH_2_-CO- | L | D | T | L | L | S | L | Q | L | I | D |  | 1395 | [19] |
| Tensin | CH_3_-(CH_2_)_6_-CHOH-CH_2_-CO- | L | D | T | L | L | S | L | Q | L | I | E |  | 1409 |
